# Impact of first-line chemoimmunotherapy with or without radiotherapy on the prognosis of patients with locally advanced or metastatic esophageal squamous cell carcinoma: a multicenter, real-world, retrospective cohort study from China (NCT06478355)

**DOI:** 10.3389/fimmu.2025.1633930

**Published:** 2025-07-28

**Authors:** Xinyi Liu, Jingyuan Wen, Yaowen Zhang, Chenyu Wang, Yatian Liu, Pudong Qian, Jianzhong Cao, Qing Hou, Yuanji Xu, Zhongmei Lin, Xianghua Ye, Min Hou, Yan Gui, Lulu Wang, Wei Zhou, Zhimin Zeng, Yaqi Song, Honglei Luo, Jiahua Lv, Wenbin Shen

**Affiliations:** ^1^ Department of Radiation Oncology, Hebei Medical University Fourth Hospital, Shijiazhuang, China; ^2^ Department of Radiation Oncology, Anyang Tumor Hospital, The Affiliated Anyang Tumor Hospital of Henan University of Science and Technology, Anyang, China; ^3^ Department of Radiation Oncology, Nanjing Medical University Affiliated Cancer Hospital & Jiangsu Cancer Hospital & Jiangsu Institute of Cancer Research, Nanjing, China; ^4^ Department of Radiation Oncology, Shanxi Province Cancer Hospital, Cancer Hospital Affiliated to Shanxi Medical University, Taiyuan, China; ^5^ Department of Radiation Oncology, Clinical Oncology School of Fujian Medical University, Fujian Cancer Hospital, Fuzhou, China; ^6^ Department of Radiation Oncology, The First Affiliated Hospital, Zhejiang University School of Medicine, Hangzhou, China; ^7^ Department of Oncology, Affiliated Hospital of North Sichuan Medical College, Nanchong, China; ^8^ Department of Radiation Oncology, Chongqing University Cancer Hospital, Chongqing, China; ^9^ Department of Oncology, The Second Affiliated Hospital of Nanchang University, Nanchang, China; ^10^ Department of Radiation Oncology, Huai’an First People’s Hospital, Huai’an, China; ^11^ Department of Radiotherapy, Sichuan Clinical Research Center for Cancer, Sichuan Hospital Cancer & Institute, Sichuan Cancer Center, University of Electronic Science and Technology of China, Chengdu, China

**Keywords:** esophageal squamous cell carcinoma, radiotherapy, immunotherapy, chemotherapy, real-world data

## Abstract

**Background:**

Chemotherapy combined with immunotherapy has already become the standard first-line treatment for advanced or metastatic esophageal squamous cell carcinoma (ESCC), whereas there are no satisfying overall survival (OS) and progression-free survival (PFS). This research aims to evaluate whether first-line chemoimmunotherapy combined with radiotherapy (RT) improves outcomes and safety in patients who suffer from locally advanced and metastatic ESCC.

**Methods:**

A total of 664 patients who suffer from locally advanced or metastatic ESCC going through first-line chemoimmunotherapy with or without radiotherapy at China’s 11 large cancer centers from Jan. 2019 to Dec. 2022 were retrospectively explored. Each patient received first-line chemoimmunotherapy, and the specific program was determined by the investigator. Regarding the radiotherapy group, each patient went through radiotherapy with a dose of ≥30 Gy to the primary lesion. Through utilizing the log-rank test, Kaplan-Meier survival curves were set up and then compared. The research carried out prognostic analysis by harnessing the univariate and multivariate Cox proportional hazards regression models. To find out patient characteristics and treatment patterns related to treatment responses, we also conducted subgroup analyses. The possible biases were minimized through performing the propensity score matching (PSM). This trial has been registered at ClinicalTrials.gov (NCT06478355, Registration date: June 22, 2024).

**Results:**

The research enrolled 664 patients in total, of which 438 received radiation therapy (ICRT group) and 226 received immunotherapy combined with chemotherapy alone (ICT group). In the overall cohort, the median follow-up was 37.0 months (IQR: 35.7-38.3). Compared to those in the ICT group, the median OS and median PFS in the ICRT group were significantly longer (mOS,33 versus 20 months, P < 0.001;mPFS, 15 versus 12 months, P < 0.001). To reduce the effect of bias, the two groups went through a 1:1 PSM analysis. The study assessed 334 patients, in which a total of 167 patients were evaluated in every subgroup. The analysis demonstrated that adding radiotherapy significantly improved the median OS (mOS, 34 versus 20 months, P=0.015) and PFS (mPFS, 16 versus 12 months, P=0.008), consistent with the pre-match results. According to the multivariate COX regression analysis, radiotherapy served as one of the independent prognostic factors that impact OS (HR=0.67,95%CI:0.50-0.89, P=0.006) and PFS (HR=0.68,95%CI:0.53-0.89, P=0.004). There were greatly prolonged both OS (HR=0.58,95%CI:0.41-0.81, P=0.002) and PFS(HR=0.61,95%CI:0.44-0.82, P=0.001) after radiotherapy within patients that just had regional lymph node metastasis. There was no benefit in OS(P=0.780) or PFS(P=0.880) within patients that had distant organ metastases. In addition, concerning patients not going through immune maintenance therapy (number of immune cycles>6), radiotherapy significantly reduced not only mortality (HR=0.66,95%CI:0.49-0.90, P=0.009) but also recurrence (HR=0.72,95%CI:0.54-0.97, P=0.028). In terms of security, ICRT group esophagitis (22.8% versus 3.6%; P<0.001), esophageal fistula (5.4% versus 0.0%; P=0.003), and pneumonia (10.8% versus 3.0%;P=0.008) all exhibited a higher incidence. Grade 3–4 pneumonia incidence was not enhanced by radiotherapy (1.8% versus 0.6%; P=0.623).

**Conclusion:**

According to the research, adding radiotherapy into systemic chemotherapy integrated with immune checkpoint inhibitors significantly improves the prognosis of patients in China who suffer from locally advanced or metastatic esophageal squamous cell carcinoma. There is safe combined treatment, and the treatment-related adverse effects are manageable. However, large randomized controlled trials need to be carried out to further confirm those results.

**Clinical trial registration:**

https://clinicaltrials.gov/study/NCT06478355, identifier NCT06478355.

## Introduction

Esophageal cancer is the most common cause of death all over the world, ranking seventh in incidence and sixth in mortality (1). In China, esophageal cancer is primarily esophageal squamous cell carcinoma (2). Anti-PD-1 antibodies combined with chemotherapy have already become the standard first-line treatment for patients with advanced or metastatic ESCC, which exhibited longer overall survival (OS) and progression-free survival (PFS) than chemotherapy alone, whereas showcased a therapeutic effect (PFS of about 6 months, and OS about 15 months) not being satisfied (3–6). Radiation therapy is widely used in all stages of esophageal cancer. As can be found in various studies, chemotherapy integrated with radiotherapy enhanced not only the local control rate but also the survival rate of patients who suffer from esophageal cancer than chemotherapy alone or radiotherapy alone (7–10). Following the KEYNOTE-001 study showing that immunotherapy has a better response in patients before going through radiation therapy, integrated radiation therapy and immunotherapy has received a lot of attention (11). In the combination of radiotherapy and immunotherapy, a good effect was identified in not only enhancing the clinical efficacy and prognosis but also strengthening the immune response to the tumor. It can synergistically enhance the antitumor effect by direct lethal effect and promoting pro-inflammatory tumor microenvironment (12, 13). In some retrospective research, it has been found that such a new treatment strategy could generate great survival benefits within locally advanced ESCC (14–16). However, research on locally advanced and metastatic ESCC radiotherapy combined with chemotherapeutic immunotherapy is limited, and there was also no clear safety and efficacy of the initial treatment with radiotherapy. As a result, a multicenter and retrospective analysis was carried out to assess both the safety and efficacy of immunochemotherapy integrated with radiotherapy versus only immunochemotherapy as the first-line treatment for patients that suffer from locally advanced and metastatic esophageal squamous cell carcinoma.

## Materials and methods

### Patients

This study was a multicenter, retrospective, and cohort study conducted at 11 large cancer centers in China (ClinicalTrials.gov NCT06478355. Registration date: June 22, 2024. The clinical trial was registered after the initiation of data collection; however, retrospective registration is considered acceptable for observational studies). Winning approval from the Fourth Hospital of Hebei Medical University’s ethics committee, the research was carried out according to the principles of the Declaration of Helsinki. Below was the primary entry criteria: (1) age that was equal to and above 18; (2) the esophageal tumor was confirmed by bite histopathology as squamous cell carcinoma; (3) the initial diagnosis of stage II-IV following the eighth edition of the cancer staging system of the American Joint Committee on Cancer (AJCC) cancer staging system; (4) the performance status (PS) of the Eastern Cooperative Oncology Group (ECOG) that was equal to or above 2, in which there was enough organ function; (5) more than two cycles of chemotherapy and more than two cycles of immunotherapy were received, in which first-line therapy went through or did not go through RT; and (6) the radiotherapy group’s patients went through ≥ 30Gy dose of radiation to the primary lesion. The research also excluded patients having gone through surgery and those who had incomplete medical records were excluded. Informed consent was abandoned due to the study is retrospective.

### Treatment

The two groups’ patients went through platinum-based chemotherapy, administered every three weeks until 4–6 cycles of chemotherapy were completed, or disease progression or unacceptable toxic side effects occurred. In addition, the specific chemotherapy protocols are formulated by the doctor in charge and mainly encompass paclitaxel + platinum or fluorouracil + platinum.

Anti-PD-1 antibodies taken as systemic immunotherapy mainly consisted of sintilimab, camrelizumab, tislelizumab, and pembrolizumab administered per three weeks till the emergence of an intolerable toxic reaction or the disease progression, or for one to two years. Immune maintenance therapy refers to receiving > 6 cycles of immunotherapy.

All patients in the ICRT group received 6–8 MeV X-ray that employed the volume-modulated arc therapy (VMAT), intensity-modulated radiotherapy (IMRT), and tomotherapy (TOMO) techniques. A total of 438 patients received radiotherapy, of which 31 received palliative treatment (dose 30-46.8 Gy, in10–26 fractions) and 407 received conventional radical treatment (dose 50–66 Gy, in 25–33 fractions), respectively. The median equivalent dose of 2 Gy fractions (EQD2) is 70.09 Gy (range:36-79.21Gy) through leveraging the linear-quadratic model with α/β = 10 Gy. As for the gross tumor volume (GTV), the primary esophageal tumors are involved, and the regional lymph nodes are also included, which encompassed or not encompassed distant metastatic lymph nodes, identified by PET/CT, CT, and gastroscopy. The clinical target volume (CTV) was 0.5-1cm outward expansion of GTV and 3cm outward expansion of upper and lower boundaries. The planning target volume (PTV) refers to the CTV expansion of 0.5-1cm. In the radiation field, the non-regional lymph nodes, which can be supraclavicular, retroperitoneal and paraaortic, are likely to be involved. According to the physical conditions and personal preferences of the patient, the decision to administer radiotherapy for distant organ metastases is realized through the attending physician.

### Statistical analysis

OS, safety and PFS all served as the main endpoints of this study. To be specific, OS refers to the time from the start of immunotherapy to the death of the patient or the final follow-up. PFS refers to the time from the immunotherapy initiation to the progression of the disease of the patient or the final follow-up. Through harnessing the t-test or the Chi-square test, basic clinical features of the patients within ICRT and ICT groups were compared, and survival curves were constructed by employing the Kaplan-Meier method and were then compared through utilizing the log-rank test. To carry out the univariate and multivariate analysis, this research harnessed the Cox proportional hazard regression model. The multivariate analysis consisted of the variables that had P < 0.05 in the univariate analysis. The independent prognostic factors for OS or PFS referred to those variables which remained P < 0.05 within the multivariate analyses. To minimize the possible bias, this research employed 1:1 PSM to match ICRT and ICT groups’ patients, in which the caliper value is 0.02 and the matching ratio is 1. R (version 4.4.2) was utilized in each statistical analysis of the research.

## Results

### Clinical characteristics

**Figure 1:**
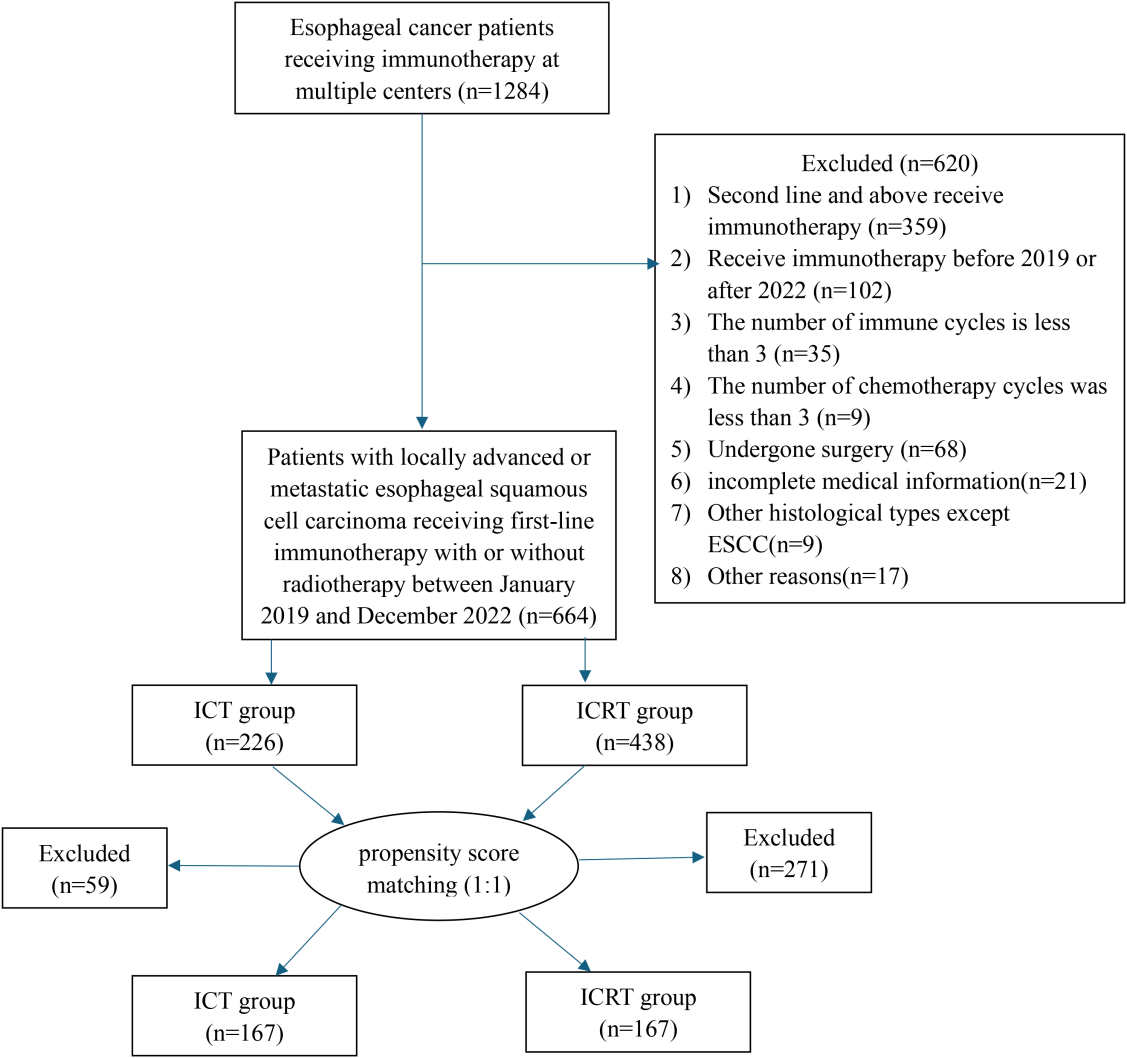
Flow chart of patient inclusion.

The study included 664 eligible patients with ESCC who went through first-line immunotherapy from 2019 to 2022. We divided patients into ICRT and ICT groups depending on whether they went through the radiotherapy. 438 people were involved in the ICRT group, while 226 people were involved in the ICT group (**Figure 1**). **Table 1** shows the patients’ clinical characteristics. Between both groups, liver metastasis, tumor location, bone metastasis, lung metastasis, and maintenance therapy all showed great differences. As for patients that had tumors within cervical and upper-thoracic, there was a higher percentage of ICRT group (cervical, 0.9% versus 6.6%, <0.001; upper-thoracic, 22.1% versus 30.1%, <0.001), lung metastases (yes, 20.4% versus 13.0%, <0.001), liver metastases (yes, 12.4% versus 2.7%, <0.001), bone metastases (yes, 8.8% versus 3.7%, 0.009), and maintenance therapy (yes, 21.7% versus 30.4%, 0.022). Patients who had received radiation seemed to be more willing to receive immune-maintenance therapy.

**Table 1:** Characteristics of patients before and after propensity score matching.

Items	Before matching (n = 664)			After matching (n = 334)		
	ICRT (N=438)	ICT (N=226)	*P*	ICRT (N=167)	ICT (N=167)	*P*
Sex			1.000			0.804
Male	330 (75.3%)	170 (75.2%)		121 (72.5%)	124 (74.3%)	
Female	108 (24.7%)	56 (24.8%)		46 (27.5%)	43 (25.7%)	
Age			0.470			0.910
<70	308 (70.3%)	152 (67.3%)		105 (62.9%)	107 (64.1%)	
≥70	130 (29.7%)	74 (32.7%)		62 (37.1%)	60 (35.9%)	
ECOG			1.000			0.733
0-1	386 (88.1%)	199 (88.1%)		149 (89.2%)	146 (87.4%)	
2	52 (11.9%)	27 (11.9%)		18 (10.8%)	21 (12.6%)	
Tumor location			<0.001			0.672
Cervical	29 (6.6%)	2 (0.9%)		1 (0.6%)	0 (0.0%)	
Upper-thoracic	132 (30.1%)	50 (22.1%)		39 (23.4%)	36 (21.6%)	
Middle-thoracic	179 (40.9%)	107 (47.3%)		85 (50.9%)	83 (49.7%)	
Lower-thoracic	98 (22.4%)	67 (29.6%)		42 (25.1%)	48 (28.7%)	
cT			0.821			0.476
T1-2	73 (16.7%)	40 (17.7%)		27 (16.2%)	33 (19.8%)	
T3-4	365 (83.3%)	186 (82.3%)		140 (83.8%)	134 (80.2%)	
cN			0.096			0.598
N0	51 (11.6%)	14 (6.2%)		8 (4.8%)	13 (7.8%)	
N1	156 (35.6%)	76 (33.6%)		59 (35.3%)	57 (34.1%)	
N2	174 (39.7%)	105 (46.5%)		77 (46.1%)	70 (41.9%)	
N3	57 (13.0%)	31 (13.7%)		23 (13.8%)	27 (16.2%)	
Lung metastases			<0.001			1.000
No	381 (87.0%)	180 (79.6%)		153 (91.6%)	153 (91.6%)	
Yes	57 (13.0%)	46 (20.4%)		14 (8.4%)	14 (8.4%)	
Liver metastases			<0.001			1.000
No	426 (97.3%)	198 (87.6%)		161 (96.4%)	161 (96.4%)	
Yes	12 (2.7%)	28 (12.4%)		6 (3.6%)	6 (3.6%)	
Bone metastases			0.009			0.619
No	422 (96.3%)	206 (91.2%)		160 (95.8%)	157 (94.0%)	
Yes	16 (3.7%)	20 (8.8%)		7 (4.2%)	10 (6.0%)	
Brain metastases			0.359			1.000
No	431 (98.4%)	225 (99.6%)		166 (99.4%)	166 (99.4%)	
Yes	7 (1.6%)	1 (0.4%)		1 (0.6%)	1 (0.6%)	
Maintenance therapy			0.022			1.000
No	305 (69.6%)	177 (78.3%)		130 (77.8%)	129 (77.2%)	
Yes	133 (30.4%)	49 (21.7%)		37 (22.2%)	38 (22.8%)	

To reduce the impact of bias, lung metastasis, sex, age, ECOG, bone metastasis, tumor location, cT, cN, brain metastasis, maintenance therapy, and liver metastasis, were all analyzed 1:1 for PSM between the two groups. Results showed that the 334 patients should be explored in the future, with 167 people per group. All covariates had p-values > 0.05 after matching, indicating a balance of each variable of both groups.

After PSM, the median number of immune cycles for both groups of patients was 4 cycles (IQR: 2–6 for both). The median number of chemotherapy cycles was 4 (IQR: 3-5; 3-6). In addition, 53.9% and 23.4% of the patients in the ICRT group before and after PSM received induction immunotherapy and concurrent immunotherapy (**Supplementary Table 1**). In the ICRT group, the median dose for tumors was 56.5Gy (IQR: 50.4-60.0). The irradiation ratio and dose for metastatic foci are detailed in **Supplementary Table 2**. Among the patients with only regional lymph node metastasis, 100% received radiotherapy for both the primary and metastatic lesions. Among patients with only non-regional lymph node metastasis, 91.3% and 92.3% received radiotherapy to the primary and metastatic lesions, respectively.

### Survival outcomes

**Figure 2:**
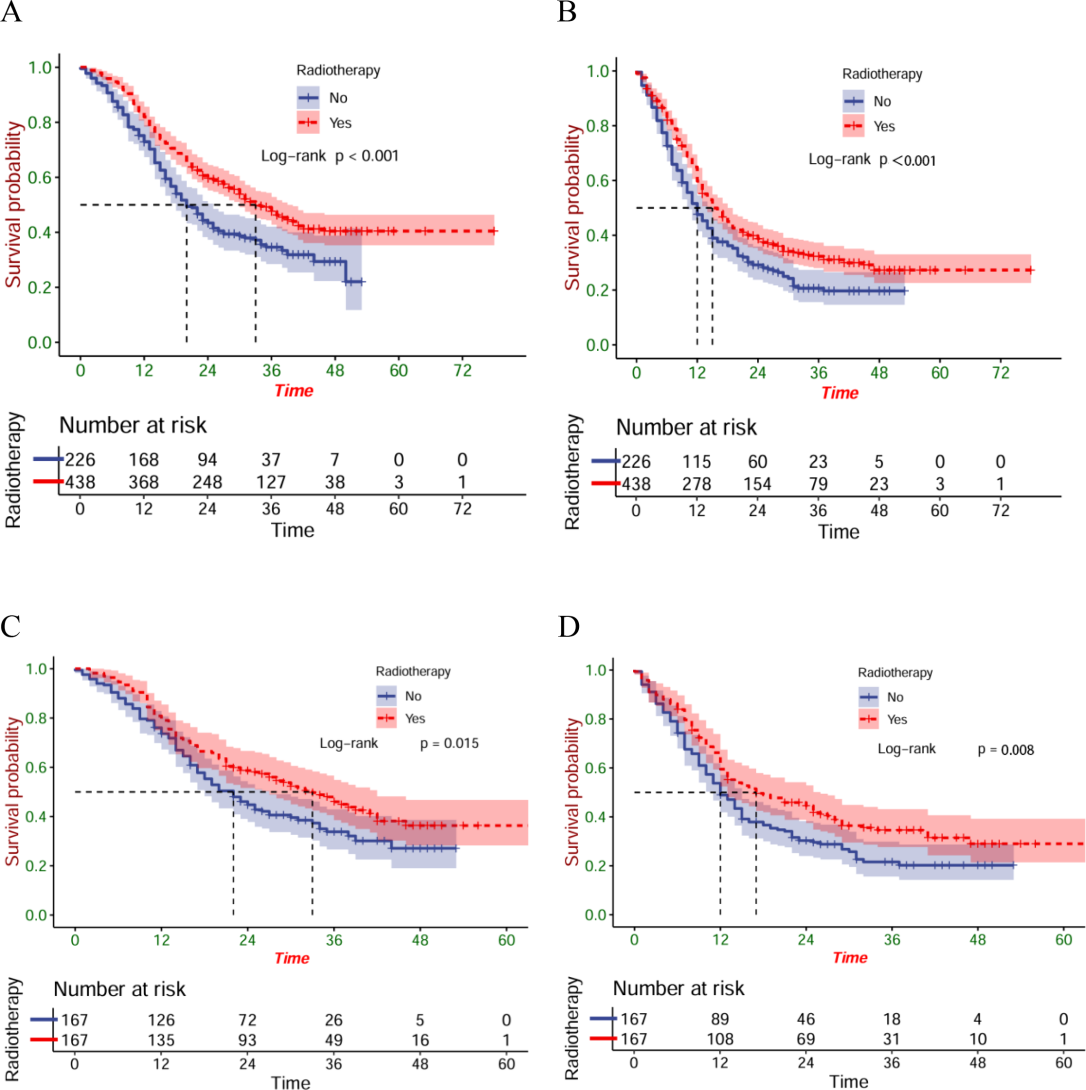
The OS **(A)** and PFS **(B)** of the entire cohort; the OS **(C)** and PFS **(D)** of the matched cohort.

Before the PSM, 37.0 months was established as each patient’s median follow-up time (IQR: 35.7-28.3). Compared with patients in the ICT group, greatly longer OS (P<0.001) and PFS (P<0.001) were found in patients within the ICRT group (**Figures 2A, B**). After PSM, there were better OS (P=0.015) (**Figure 2C**) and PFS (P=0.008) (**Figure 2D**) of the patients of the ICRT group compared to the ICT group. A total of 92 patients (55.1%) had died in the ICRT group and 105 patients (62.9%) in the ICT group by the follow-up date. The corresponding mOS were 34 months (95%CI:27.0-41.0) and 20 months (95%CI:17.7-26.3), respectively, and the mPFS was 16 months (95%CI:11.6-20.4) and 12 months (95%CI:9.8-14.2), separately.

### Univariate and multivariate COX regression analyses

Since patients that have distant metastases within the two groups are lower, metastatic sites were combined into four categories for univariate and multivariate analyses, which are regional lymph node metastasis, distant organ metastasis, non-regional lymph node metastasis, and the presence of all types.

For all patients before PSM, univariate analysis showed that ECOG, cT, cN, metastatic sites, maintenance therapy, and radiotherapy were significant factors affecting OS. The multivariate Cox regression further used all the significant factors within the univariate Cox regression analysis. We found that the independent prognostic factors that impacted OS consisted of the ECOG, cT, cN, and maintenance therapy. At the same time, we found that there was no obvious P value although radiotherapy tended to decrease the death risk (HR=0.80,95%CI:0.64-1.00, P=0.053) (**Table 2**). Similarly, as can be found in the results of multivariate analysis, independent prognostic factors affecting patients’ PFS involved both cN and metastatic sites, but radiotherapy did not have significant significance (**Table 3**).

**Table 2:** Univariate and multivariate Cox regression analyses for OS for ESCC patients before propensity score matching.

Characteristics	Univariate		Multivariate	
	HR (95% CI)	*P-*value	HR (95% CI)	*P-*value
Sex			Not selected	
Male	Reference			
Female	0.92 (0.73-1.17)	0.510		
Age			Not selected	
<70	Reference			
≥70	1.19 (0.96-1.47)	0.110		
ECOG				
0-1	Reference		Reference	
2	1.66 (1.25-2.21)	**<0.001**	1.69 (1.25-2.29)	**0.001**
Tumor location			Not selected	
Cervical	Reference			
Upper-thoracic	0.75 (0.46-1.23)	0.250		
Middle-thoracic	1.03 (0.64-1.65)	0.920		
Lower-thoracic	1.07 (0.65-1.76)	0.780		
**cT**				
T1-2	Reference		Reference	
T3-4	1.55 (1.16-2.07)	**0.003**	1.46 (1.08-1.96)	**0.013**
**cN**				
N0	Reference		Reference	
N1	1.84 (1.15-2.94)	**0.011**	1.86 (1.17-2.98)	**0.009**
N2	2.35 (1.48-3.74)	**<0.001**	2.05 (1.29-3.28)	**0.003**
N3	2.74 (1.66-4.53)	**<0.001**	2.56 (1.54-4.28)	**<0.001**
Metastatic sites			Not selected	
Regional lymph nodes	Reference			
Non-regional lymph nodes only	1.080 (0.77-1.41)	0.670	1.04 (0.74-1.47)	0.820
Distant organ only	1.30 (1.00-1.69)	0.053	1.21 (0.91-1.60)	0.190
Both	1.65 (1.14-2.38)	**0.007**	1.39 (0.90-2.14)	0.140
Maintenance therapy			Not selected	
No	Reference		Reference	
Yes	0.54 (0.43-0.68)	**<0.001**	0.52 (0.41-0.66)	**<0.001**
Radiotherapy				
No	Reference		Reference	
Yes	0.69 (0.56-0.85)	**0.001**	0.80 (0.64-1.00)	0.053

Significant values (P < 0.05) are highlighted in bold.

**Table 3:** Univariate and multivariate Cox regression analyses for PFS for ESCC patients before propensity score matching.

Characteristics	Univariate		Multivariate	
	HR (95% CI)	*P-*value	HR (95% CI)	*P-*value
Sex			Not selected	
Male	Reference			
Female	0.81 (0.66-1.01)	0.058		
Age			Not selected	
<70	Reference			
≥70	1.01 (0.84-1.23)	0.890		
ECOG			Not selected	
0-1	Reference			
2	1.21 (0.93-1.56)	0.150		
Tumor location			Not selected	
Cervical	Reference			
Upper-thoracic	0.78 (0.51-1.19)	0.250		
Middle-thoracic	0.90 (0.60-1.35)	0.620		
Lower-thoracic	1.14 (0.75-1.72)	0.550		
**cT**			Not selected	
T1-2	Reference			
T3-4	1.26 (0.99-1.59)	0.057		
cN				
N0	Reference		Reference	
N1	1.53 (1.06-2.21)	**0.023**	1.50 (1.03-2.18)	**0.034**
N2	1.98 (1.38-2.84)	**<0.001**	1.91 (1.32-2.76)	**0.001**
N3	2.04 (1.35-3.08)	**0.001**	2.04 (1.34-3.08)	**0.001**
Metastatic sites				
Regional lymph nodes	Reference		Reference	
Non-regional lymph nodes only	1.38 (1.08-1.76)	**0.011**	1.31 (1.02-1.68)	**0.034**
Distant organ only	1.48 (1.16-1.90)	**0.002**	1.40 (1.08-1.81)	**0.010**
Both	1.62 (1.11-2.37)	**0.013**	1.41 (0.95-2.09)	0.085
Maintenance therapy			Not selected	
No	Reference			
Yes	0.84 (0.69-1.02)	0.082		
Radiotherapy				
No	Reference		Reference	
Yes	0.74 (0.61-0.89)	**0.002**	0.83 (0.68-1.02)	0.072

Significant values (P < 0.05) are highlighted in bold.

Univariate analysis after PSM showed that obvious factors that exerted an impact on OS involved age, tumor location, cT, cN, maintenance therapy, and radiotherapy, and that the obvious factors that exerted an impact on PFS included tumor location, cN, maintenance therapy, and radiotherapy. According to the multivariate analysis, independent factors affecting OS and PFS consisted of cN, maintenance therapy, and radiotherapy. Matched population analysis showed that patients could recover from radiotherapy (OS, HR=0.67,95%CI:0.50-0.89, P=0.006; PFS, HR=0.68, 95%CI:0.53-0.89, P=0.004) and that immune maintenance therapy was beneficial (OS, HR=0.44,95%CI:0.30-0.64, P<0.001; PFS, HR=0.72,95%CI:0.52-0.99, p=0.045) (**Table 4**).

**Table 4:** Univariate and multivariate Cox regression analyses for OS and PFS for ESCC patients after propensity score matching.

Characteristics	OS				PFS			
	Univariate		Multivariate		Univariate		Multivariate	
	HR (95% CI)	*P*	HR (95% CI)	*P*	HR (95% CI)	*P*	HR (95% CI)	*P*
Sex								
Male	0.96 (0.70-1.31)	0.783			1.16 (0.86-1.56)	0.335		
Female								
Age								
<70								
≥70	1.34 (1.01-1.77)	**0.045**	1.22 (0.92-1.63)	0.172	1.16 (0.89-1.51)	0.261		
ECOG								
0-1								
2	1.15 (0.76-1.75)	0.513			1.08 (0.73-1.60)	0.709		
Tumor location								
Cervical								
Upper-thoracic	0.08 (0.01-0.60)	**0.014**	0.14 (0.02-1.04)	0.054	0.11 (0.01-0.80)	**0.029**	0.13 (0.02-0.96)	**0.046**
Middle-thoracic	0.10 (0.01-0.71)	**0.022**	0.15 (0.02-1.11)	0.063	0.12 (0.02-0.88)	**0.037**	0.14 (0.02-1.00)	0.050
Lower-thoracic	0.11 (0.01-0.80)	**0.030**	0.17 (0.02-1.33)	0.092	0.16 (0.02-1.21)	0.076	0.19 (0.02-1.38)	0.100
cT								
T1-2								
T3-4	1.65 (1.11-2.45)	**0.013**	1.46 (0.97-2.20)	0.069	1.37 (0.97-1.93)	0.077		
cN								
N0								
N1	3.06 (1.23-7.60)	**0.016**	3.01 (1.21-7.49)	**0.018**	2.09 (1.05-4.17)	**0.036**	2.04 (1.02-4.07)	**0.044**
N2	3.76 (1.53-9.25)	**0.004**	3.17 (1.28-7.86)	**0.013**	2.65 (1.34-5.24)	**0.005**	2.48 (1.25-4.91)	**0.009**
N3	4.47 (1.75-11.45)	**0.002**	4.39 (1.70-11.34)	**0.002**	2.97 (1.43-6.16)	**0.003**	2.89 (1.38-6.02)	**0.005**
Metastatic sites								
Regional lymph nodes								
Non-regional lymph nodes only	1.18 (0.75-1.87)	0.475			1.17 (0.78-1.76)	0.448		
Distant organ only	1.15 (0.77-1.73)	0.496			1.05 (0.71-1.56)	0.805		
Both	1.39 (0.77-2.50)	0.277			1.56 (0.91-2.70)	0.109		
Maintenance therapy								
No								
Yes	0.45 (0.31-0.66)	**<0.001**	0.44 (0.30-0.64)	**<0.001**	0.71 (0.51-0.97)	**0.033**	0.72 (0.52-0.99)	**0.045**
Radiotherapy								
No								
Yes	0.68 (0.51-0.90)	**0.007**	0.67 (0.50-0.89)	**0.006**	0.70 (0.54-0.91)	**0.007**	0.68 (0.53-0.89)	**0.004**

Significant values (P < 0.05) are highlighted in bold.

### Subgroup analysis

**Figure 3:**
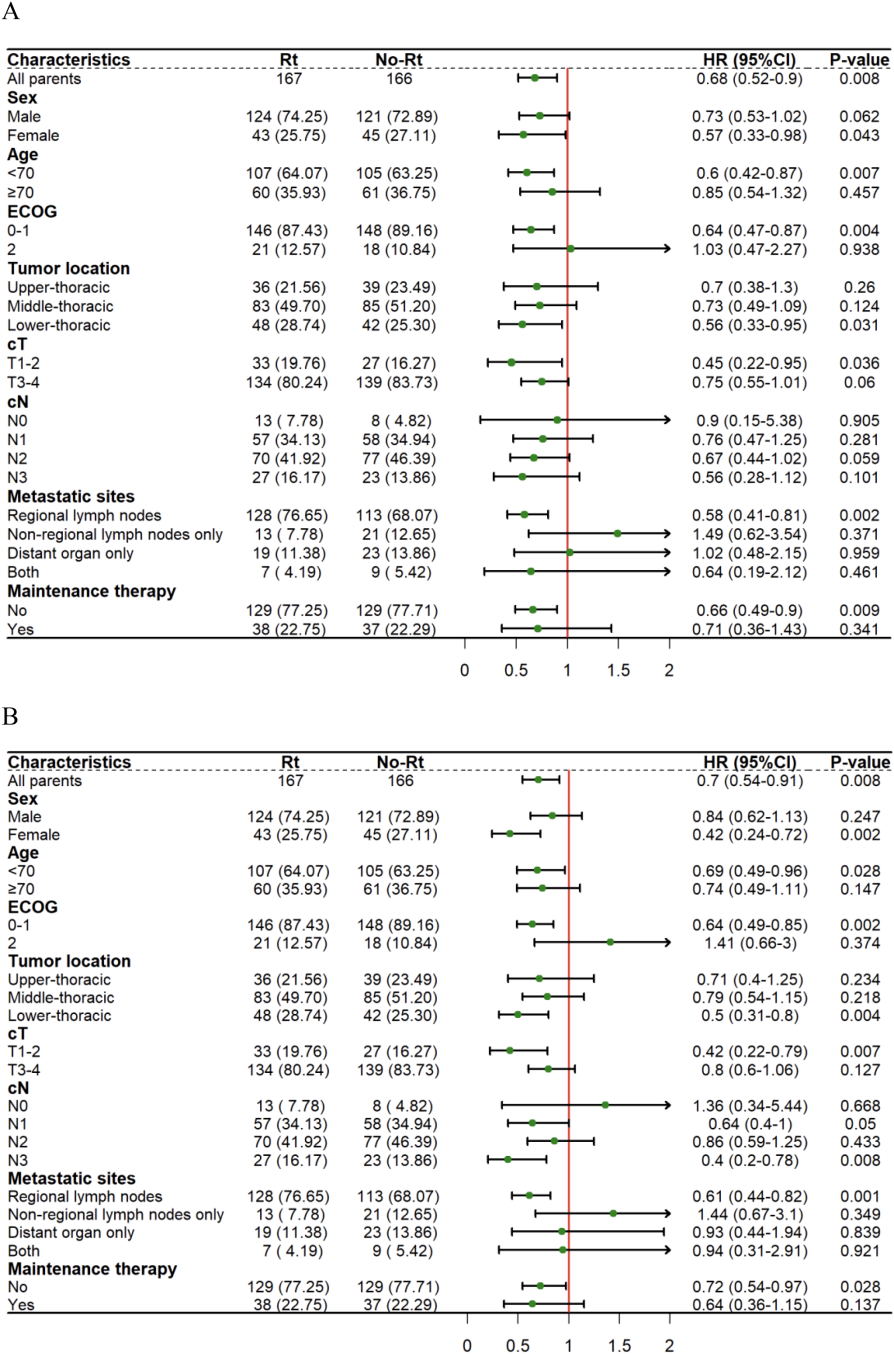
Forest plots show factors associated with OS **(A)** and PFS **(B)** of thoracic segment esophageal carcinoma within the matched cohort.

An exploratory subgroup analysis was then performed between the ICRT and ICT groups. Since only one patient in the two groups had tumor location in the cervical after matching and could not be compared, subgroup analysis excluded such a patient. As for the OS subgroup analysis, the RT group included female patients (HR=0.57, 95%CI:0.33-0.98, P=0.043), patients < 70 years old (HR=0.60, 95%CI:0.42-0.87, P=0.007), patients that had the ECOG score of 0-1 (HR=0.64, 95%CI:0.47-0.87, P=0.004), and patients that had tumor location in the lower thoracic segment (HR=0.56, 95%CI:0.33-0.95, P=0.031), patients with clinical stage T1-2 (HR=0.45, 95%CI:0.22-0.95, P=0.036), patients that just had regional lymph node metastasis (HR=0.58, 95%CI:0.41-0.81, P=0.002) and patients having not to go through immune-maintenance therapy (HR=0.66, 95%CI:0.49-0.90, P=0.009) for a significantly longer OS (**Figure 3A**). At the same time, within the PFS subgroup analysis, the RT group included female patients (HR=0.42,95%CI:0.24-0.72, P=0.002), patients < 70 years old (HR=0.69, 95%CI:0.49-0.96, P=0.028), and the ECOG score of the patients ranged from 0 to 1 (HR=0.64, 95%CI:0.49-0.85, P=0.002), and the lower thoracic segment of the patient’s tumor location (HR=0.50, 95%CI:0.31-0.80, P=0.004), the patient’s clinical stage was T1-2 (HR=0.42, 95%CI:0.22-0.79, P=0.007), the clinical stage of the patient lied in N3 (HR=0.40,95%CI:0.20-0.78, P=0.008), and the patient only had regional lymph node metastasis (HR=0.61, 95%CI:0.44-0.82, P=0.001), and patients who did not receive immune-maintenance therapy (HR=0.72, 95%CI:0.54-0.97, P=0.028) had significantly prolonged PFS (**Figure 3B**).

**Figure 4:**
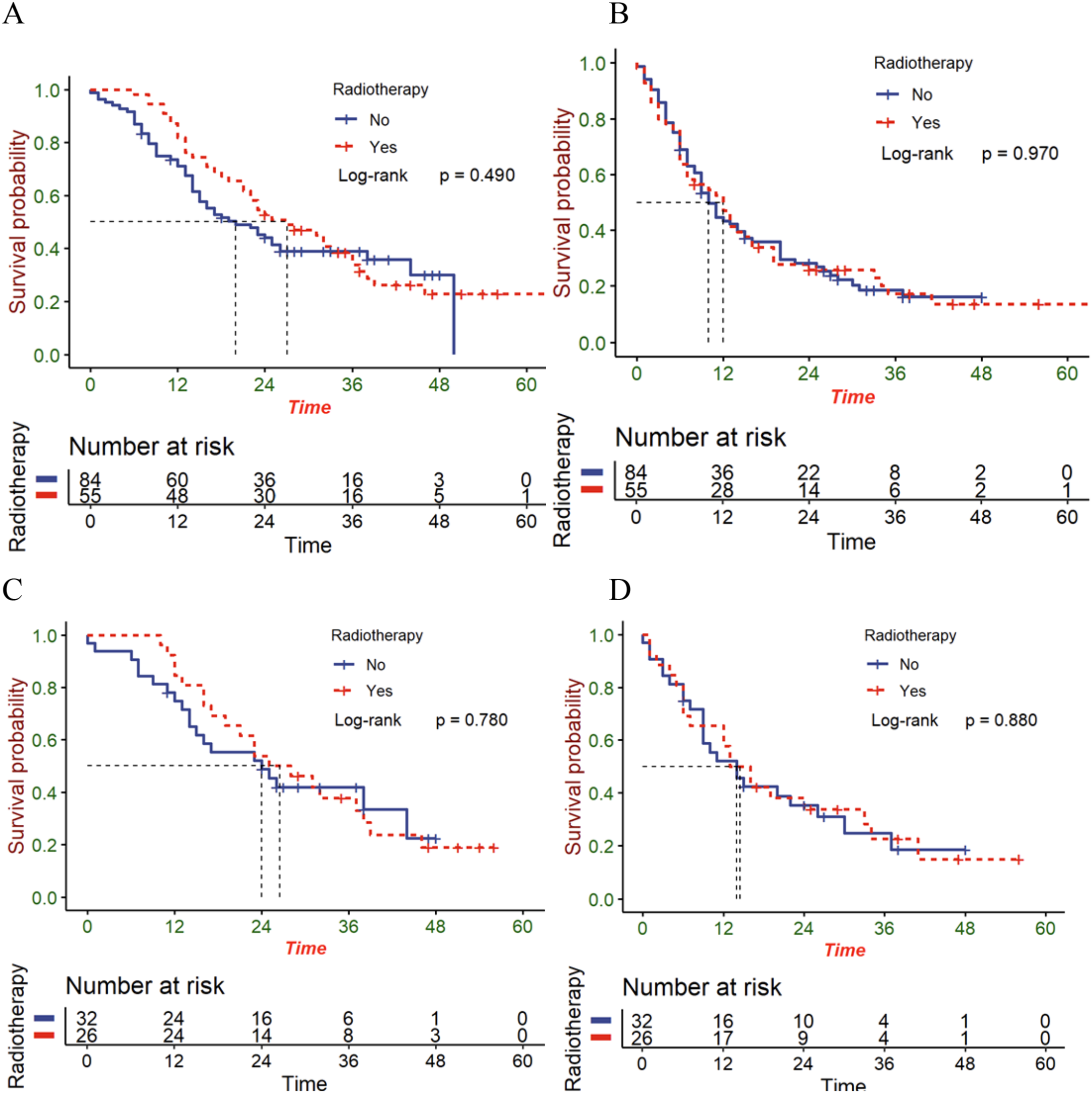
The OS **(A)** and PFS **(B)** in patients with distant organ involvement in the entire cohort; the OS **(C)** and PFS **(D)** in patients with distant organ involvement in the matched cohort.

To explore the impacts exerted by radiotherapy on different metastatic sites, survival analyses were then carried out on patients who have distant organ involvement. Unfortunately, patients with distant organ involvement, whether before or after matching showed no statistical differences between the OS (**Figures 4A, C**) and the PFS (**Figures 4B, D**). Perhaps, it is essential to cautiously treat patients that have metastatic esophageal squamous cell carcinoma.

**Figure 5:**
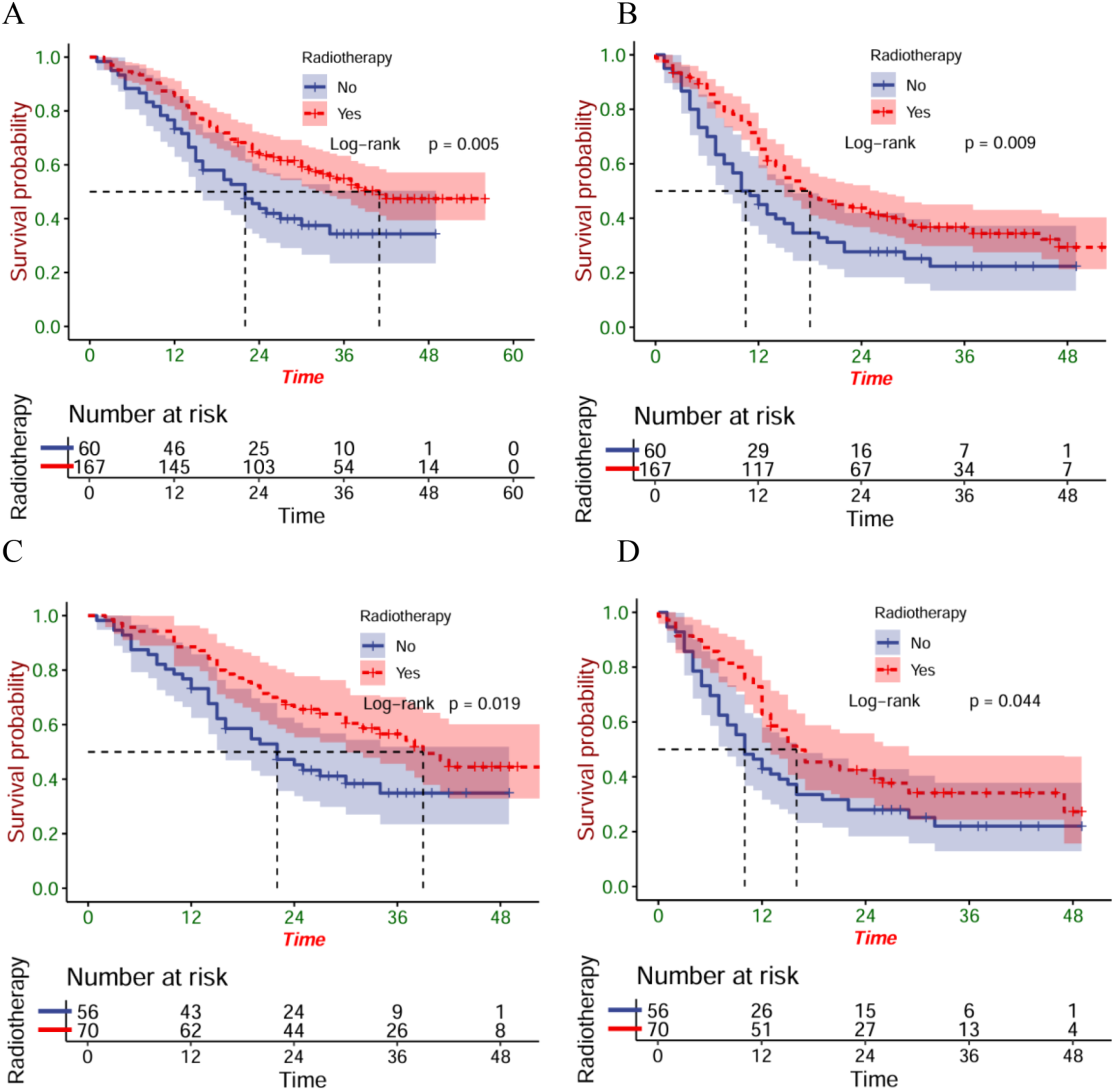
The OS **(A)** and PFS **(B)** of stage III patients within the entire cohort; the OS **(C)** and PFS **(D)** of stage III patients within the matched cohort.

Given that radiotherapy combined with immunotherapy is mainly employed in patients with esophageal cancer with clinical stage III, the effect of radiotherapy in patients with stage III was further analyzed. In the entire cohort, 167 stage III patients received radiotherapy and 60 received chemoimmunotherapy alone. For pre-matched stage III patients, the radiotherapy group exhibited better survival outcomes (mOS=22 versus 41 months; mPFS=10.5 versus 18 months) (**Figures 5A, B**). At the same time, the matched radiotherapy group showcased a better prognosis (mOS=22 versus 39 months; mPFS=10 versus 16 months) (**Figures 5C, D**).

### Safety analysis

The toxicity of the matched cohort during the follow-up period was analyzed (**Table 5**). The research graded the adverse effects (AEs) following the National Cancer Institute Common Terminology Criteria for Adverse Events, version 4.03 (NCI CTCAE v4.03). Compared to that of the ICT group, there was a greater total incidence of esophagitis per grade within the ICRT group (22.8% versus 3.6%, P<0.001). Radiotherapy increased the incidence of not only the grade 1–2 esophagitis (18.6% versus 3.6%, P<0.001) but also the grade 3–4 esophagitis (4.2% versus 0.0%, P=0.015). At the same time, radiotherapy combined with immunotherapy increased the overall incidence of pneumonia of any grade (10.8% versus 3.0%, P=0.008), while both groups showed no significant difference within the grade 3–4 pneumonia incidence (1.8% versus 0.6%, P=0.623). Within the ICRT group, a total of 9 patients developed esophageal fistula, whereas, in the ICT group, there was no esophageal fistula (5.4% versus 0.0%; P = 0.003). Besides, no obvious differences were found in the incidence of myelosuppression, immunotherapy-related dermatitis, immune-associated myositis, hypohepatia, hyperthyroidism, and hypothyroidism between ICRT and ICT groups.

**Table 5:** Acute toxicities in the 334 patients of the matched cohort.

Adverse events	No-Rt	Rt	*P*-value
	(N=167)	(N=167)	
Myelosuppression	54 (32.4%)	54 (32.4%)	1.000
Grade 1-2	39 (23.4%)	29 (17.4%)	0.221
Grade 3-4	15 (9.0%)	25 (15.0%)	0.129
Esophagitis	6 (3.6%)	38 (22.8%)	<0.001
Grade 1-2	6 (3.6%)	31 (18.6%)	<0.001
Grade 3-4	0 (0.0%)	7 (4.2%)	0.015
Pneumonia	5 (3.0%)	18 (10.8%)	0.008
Grade 1-2	4 (2.4%)	15 (9.0%)	0.009
Grade 3-4	1 (0.6%)	3 (1.8%)	0.623
Esophagostoma	0 (0.0%)	9 (5.4%)	0.003
Immunotherapy-related dermatitis	7 (4.2%)	6 (3.6%)	1.000
Hypohepatia	9 (5.4%)	7 (4.2%)	0.799
Hyperthyroidism	4 (2.4%)	3 (1.8%)	1.000
Hypothyroidism	10 (6.0%)	9 (5.4%)	1.000
Immune-associated myositis	6 (3.6%)	4 (2.4%)	0.750

## Discussion

In this multicentre retrospective study of 664 patients with untreated locally advanced and metastatic ESCC, the results show that receiving first-line immunotherapy combined with radiotherapy exhibits significant OS and PFS benefits compared to receiving first-line immunotherapy alone. Due to the inherent limitations of retrospective studies and the potential selection biases, the PSM analysis was carried out to identify RT impacts more precisely. As can be found in the PSM analysis, certain advantages can be seen in the addition of RT from the perspectives of both OS and PFS. In the multivariate Cox regression analysis, it was also underpinned that radiotherapy had an obvious relationship with better OS and PFS. The addition of radiotherapy is safe and reliable. In addition, we also investigated the effectiveness of different modes of metastasis and whether to receive immune-maintenance therapy for this regimen. Exploratory analyses showed significant survival benefits within patients who not only have just regional lymph node metastases but also those who do not go through immune-maintenance therapy. In addition, as for patients that have stage III esophageal squamous cell carcinoma, in which the main group went through first-line radioimmunotherapy, both significant OS and PFS benefits were also caused by adding radiotherapy.

In patients with advanced ESCC with chemotherapy and anti-PD-1 antibodies treatment, the OS and PFS of 12.4–17 months and 5.7-7.2 months were reported by the multiple randomized Phase III trials (3, 4, 6, 17) respectively. In this study, 20 months (95% CI: 17.7-26.3) and 12 months (95% CI: 9.8-14.2) served as the median OS and PFS of the No-RT group. Both OS and PFS were slightly higher than in the above clinical trials, which might be related to the inclusion of only about 10-20% of locally advanced patients in KEYNOTE-590 (3), ESCORT-1^st^ (4), ORIENT-15 (6), and JUPITER-06 (17) trials, compared to 68.3% (114/167) of locally advanced patients. Meanwhile, the research just consisted of patients that had esophageal squamous cell carcinoma. In addition, real-world studies have higher heterogeneity of patients and treatment regimens than RCTS.

The 34 months (95% CI: 27.0-41.0) and 16 months (95% CI: 11.6-20.4) were the median OS and PFS of the RT group. Survival data for advanced esophageal cancer receiving first-line radiotherapy combined with immunization are lacking. Large Phase III clinical trials such as KEYNOTE-975 (18) are still underway. In a retrospective study comparing combined radiotherapy with immunochemotherapy alone as first-line treatment for early treatment of advanced esophageal squamous cell carcinoma, Jiacheng Li et al. (19) found a connection between radiotherapy and better OS between 16.8 and 20.4 months. However, adding radiotherapy improved no PFS than just chemotherapy. Similar to ours, Biqi Chen (20) found adding radiotherapy enhanced not only OS but also PFS (median OS: 24.9 vs. 14.6 months, P = 0.003; Median PFS: 14.2 vs. 10.6 months, P = 0.002). Also, perhaps because our research consisted of many patients that had locally advanced disease, the median OS and PFS were better than those in the above studies. Hui-Hui Hu et al. (21) also compared the survival of first-line chemoimmunotherapy plus radiotherapy for locally advanced or metastatic esophageal squamous cell carcinoma, in which the OS and PFS were 31.8 (95% CI, 23.0-NA) months and 13.5 (95% CI, 10.4-NA) months. Compared to the research of Hui-Hui Hu et al., our median OS and PFS were slightly better. Overall, the larger sample size of our study (664 vs. 72) and the use of PSM to reduce the effect of bias clarified both the safety and efficacy of the radiation therapy within the locally advanced and metastatic ESCC.

It was seen in the subgroup survival analysis that great benefits were obtained from radiation therapy by patients who had simple regional lymph node metastases. Besides, patients who developed non-regional lymph nodes did not have a similar survival advantage. However, previous studies (20, 22) have shown that great benefits can be obtained from radiotherapy by ESCC patients who have simple non-regional lymph node metastasis. This difference may be due to the following reasons. First, despite the complicated correlation between radiotherapy and immunotherapy, there is larger damage to the immune system when the radiotherapy scope is larger, especially in the case of greater radiation doses (23). Although local tumor radiotherapy can improve tumor control in the radiological field, the distant effect is diminished as CD8+ T cells decrease when radiotherapy is used to treat the tumor primary site and lymph nodes (23). 92.9% (407/438) of the primary tumors in the research received radical radiation therapy (dose 50–66 Gy and within 25–33 fractions). Increased radiation coverage of patients with nonregional lymph node metastases may account for the difference in survival. Furthermore, patients with distant organ metastases did not gain significant survival benefits after radiotherapy, which is consistent with previous studies (19, 21). In this study, only some patients with distant organ metastasis received radiotherapy for the primary and metastatic lesions, and 5 patients only received palliative radiotherapy for primary lesions. Meanwhile, whether patients in the research had oligometastasis was not distinguished, which may be the reason why radiotherapy failed to benefit.

Also, the order in which radiotherapy and immunization were administered may account for the difference in results. Some researchers (24) believe that the immune system is more functional before radiotherapy and therefore more likely to respond to immunosuppressants. In contrast, according to the Phase II non-randomized analysis within patients that have stage III non-small cell lung cancer, combined immunotherapy with concurrent chemoradiotherapy exhibits a good anti-tumor effect and good safety profile (25). In our study, many patients not only received induction immunotherapy but also concurrent immunotherapy during radiotherapy. Only a small number of patients received simple sequential treatment and concurrent radioimmunotherapy, so further analysis was not possible.

In general, the safety of the trial conformed to before reported safety among patients who had advanced ESCC and received PD-1 inhibitors integrated with chemotherapy as the first-line treatment (3, 26, 27). Although the addition of radiotherapy enhanced esophagitis and pneumonia incidence, the incidence of grade 3–4 esophagitis was just 4.2%, and the incidence of grade 3–4 pneumonia showed no significant enhancement. Compared to that of the No-RT group, there was a larger esophageal fistula incidence within the RT group. Therefore, in the era of immunization, the radiotherapy dose and safety should be carefully selected for the esophageal cancer patients at T4 when there is no obvious evidence of benefit. However, the incidence of esophageal fistula in the RT group was only 5.4%. In summary, the overall safety profile of combination therapy in locally advanced and metastatic ESCC was considered satisfactory and well tolerated. Our research focuses on whether locally advanced and metastatic ESCC receiving first-line chemotherapy combined with immunotherapy can benefit from radiotherapy. Retrospective collection of toxicity characteristics with a limited sample size may lead to a certain degree of underestimation.

In our study, there are obvious limitations. Firstly, a large gap exists in the number of patients with advanced ESCC and metastatic ESCC. There were relatively smaller subgroups of patients that had non-regional lymph node metastasis and distant organ metastasis, and there were differences in the types of organs involved and the target and dose of radiotherapy. The non-regional lymph node metastases or distant organ metastases exhibited no survival benefits. It is necessary to further increase the sample size of patients for prospective studies and therefore explicitly explore this issue. Secondly, the study lacks further subgroup analysis of biomarkers, especially the expression levels of PD-L1, TMB or MSI. In PD-L1-positive patients, the subgroup analysis of the KEYNOTE-590 (3) study showed that ESCC patients with PD-L1 CPS≥10 benefited more than those with CPS<10. Regrettably, as less than a quarter of the patients were tested for PD-L1 status, the discussion in this study was insufficient. Further randomized phase III trials are crucial to optimize the integration of radiotherapy into initial systemic treatment regimens, clarify its therapeutic advantages, and identify patients most likely to benefit. Finally, the results may be affected by possible factors like selection bias since the research is retrospective. For instance, patients may experience variations in treatment cycles, undergo different immunotherapy and chemotherapy regimens, receive distinct radiotherapy target areas and dosage levels, and follow diverse sequencing strategies involving combinations of radiotherapy, chemotherapy, and immunotherapy. However, we had a large sample size and applied PSM to minimize the influence of various biases.

## Conclusion

According to the research, adding radiotherapy into systemic chemotherapy integrated with immune checkpoint inhibitors significantly improves the prognosis of patients in China who suffer from locally advanced or metastatic esophageal squamous cell carcinoma. There is safe combined treatment, and the treatment-related adverse effects are manageable. However, large randomized controlled trials need to be carried out to further confirm those results.

## Data Availability

The raw data supporting the conclusions of this article will be made available by the authors, without undue reservation.

## Supplementary material

Supplementary Table 1 Treatment details in two groups before and after PSM.

Supplementary Table 2 Proportion of irradiated metastatic lesions and corresponding doses in subgroups before and after PSM.
