# Supplementary material for: Impact of first-line chemoimmunotherapy with or without radiotherapy on the prognosis of patients with locally advanced or metastatic esophageal squamous cell carcinoma: a multicenter, real-world, retrospective cohort study from China (NCT06478355)
Source: Front Immunol. 2025 Jul 28;16:1633930. doi: 10.3389/fimmu.2025.1633930 (PMC12336177; doi:10.3389/fimmu.2025.1633930)
Supplement: Supplementary Table 1 — Treatment details in two groups before and after PSM. [file Table1.docx]

eTable 1. Treatment details in two groups before and after PSM.

| **Treatment** | **Before Matching (n = 664)** | | **After matching (n = 334)** | |
| --- | --- | --- | --- | --- |
|  | ICRT  (N=438) | ICT  (N=226) | ICRT  (N=167) | ICT  (N=167) |
| Induction chemotherapy (%) | 245 (55.9) | / | 94 (56.3) | / |
| Induction immunotherapy (%) | 228 (52.1) | / | 90 (53.9) | / |
| Concurrent chemotherapy (%) | 195 (44.5) | / | 65 (38.9) | / |
| Concurrent immunotherapy (%) | 123 (28.1) | / | 39 (23.4) | / |
| Consolidation chemotherapy (%) | 132 (30.1) | / | 50 (29.9) | / |
| Consolidation immunotherapy (%) | 221 (50.5) | / | 79 (47.3) | / |
| Total anti-PD-1 antibody cycles (median, IQR) | 4 (2-8) | 4 (2-6) | 4 (2-6) | 4 (2-6) |
| Camrelizumab (%) | 146 (33.3) | 111 (49.1) | 57 (34.1) | 80 (47.9) |
| Sintilimab (%) | 123 (28.1) | 79 (35.0) | 45 (26.9) | 60 (35.9) |
| Tislelizumab (%) | 65 (14.8) | 9 (4.0) | 27 (16.2) | 8 (4.8) |
| Pembrolizumab (%) | 58 (13.2) | 9 (4.0) | 21 (12.6) | 7 (4.2) |
| Toripalimab (%) | 36 (8.2) | 8 (3.5) | 14 (8.4) | 5 (3.0) |
| Others (%) | 10 (2.3) | 10 (4.4) | 3 (1.8) | 7 (4.2) |
| Total chemotherapy cycles (median, IQR) | 4 (3-6) | 4 (3-6) | 4 (3-5) | 4 (3-6) |
| TP (%) | 314 (71.7) | 147 (65.0) | 126 (75.4) | 116 (69.5) |
| PF (%) | 51 (11.6) | 32 (14.2) | 21 (12.6) | 24 (14.4) |
| Single agent (%) | 47 (10.7) | 31 (13.7) | 12 (7.2) | 14 (8.4) |
| Others (%) | 26 (5.9) | 16 (7.1) | 8 (4.8) | 13 (7.8) |

Abbreviations: TP, taxanes and platinum; PF, platinum and 5-fluorouracil.
