# Supplementary material for: Impact of first-line chemoimmunotherapy with or without radiotherapy on the prognosis of patients with locally advanced or metastatic esophageal squamous cell carcinoma: a multicenter, real-world, retrospective cohort study from China (NCT06478355)
Source: Front Immunol. 2025 Jul 28;16:1633930. doi: 10.3389/fimmu.2025.1633930 (PMC12336177; doi:10.3389/fimmu.2025.1633930)
Supplement: Supplementary Table 2 — Proportion of irradiated metastatic lesions and corresponding doses in subgroups before and after PSM. [file Table2.docx]

eTable 2. Irradiation proportion and dose for metastatic lesions in subgroups before and after PSM.

| **Metastases subgroup** | **Radiation of metastatic lesions** | | | | |
| --- | --- | --- | --- | --- | --- |
|  | **Before PSM (n=438)** | | | | |
|  | Irradiation  Proportion(%) | Dose for LNs,Gy  median (IQR) | Dose per fraction, Gy  median (range) | Dose for organs, Gy  median (IQR) | Dose per fraction, Gy  median (range) |
| Regional lymph nodes (LNs) only | 337/337(100.0) | 59.4 (50.4-60.0) | 2.0 (1.7-4.5) | / | / |
| Non-regional lymph nodes only | 42/46 (91.3) | 59.4 (50.4-60.0) | 2.0 (1.8-3.0) | / | / |
| Distant organ only | 13/46 (28.3) | / | / | 45.0 (32.0-48.0) | 3.0 (2.0-8.0) |
| Non-regional LNs combined  with distant organ | / | / | / | / | / |
| Irradation of non-regional LNs only | 7/9 (77.8) | 50.4 (50.0-55.2) | 2.0 (1.8-2.2) | / | / |
| Irradation of both | 1/9 (11.1) | 50.4 | 1.8 | 50.0 | 10.0 |
| **After PSM (n=167)** | | | | | |
| Regional lymph nodes (LNs) only | 128/128(100.0) | 59.7 (50.4-60.0) | 2.0 (1.8-2.5) | / | / |
| Non-regional lymph nodes only | 12/13 (92.3) | 56.0 (50.3-60.0) | 2.0 (1.8-3.0) | / | / |
| Distant organ only | 2/19 (10.5) | / | / | 40.0 (40.0-40.0) | 3.5 (2.0-5.0) |
| Non-regional LNs combined  with distant organ | / | / | / | / | / |
| Irradation of non-regional LNs only | 5/7 (71.4) | 50.0 (50.0-50.4) | 2.0 (1.8-2.0) | / | / |
| Irradation of both | 1/7 (14.3) | 50.4 | 1.8 | 50.0 | 10.0 |
